# Reporting of equity in observational epidemiology: A methodological review

**DOI:** 10.7189/jogh.14.04046

**Published:** 2024-03-01

**Authors:** Omar Dewidar, Ali Al-Zubaidi, Mostafa Bondok, Leenah Abdelrazeq, Jimmy Huang, Alyssa Jearvis, Lucy C Barker, Nour Elmestekawy, Elizabeth Goghomu, Tamara Rader, Janice Tufte, Regina Greer-Smith, Hugh S Waddington, Stuart G Nicholls, Julian Little, Billie-Jo Hardy, Tanya Horsley, Taryn Young, Luis Gabriel Cuervo, Melissa K Sharp, Catherine Chamberlain, Beverley Shea, Peter Craig, Daeria O Lawson, Anita Rizvi, Charles S Wiysonge, Tamara Kredo, Damian Francis, Elizabeth Kristjansson, Zulfiqar Bhutta, Alba Antequera, GJ Melendez-Torres, Tomas Pantoja, Xiaoqin Wang, Janet Jull, Janet Hatcher Roberts, Sarah Funnell, Howard White, Alison Krentel, Michael Johnson Mahande, Jacqueline Ramke, George Wells, Jennifer Petkovic, Kevin Pottie, Loveline Niba, Cindy Feng, Miriam N Nguliefem, Peter Tugwell, Lawrence Mbuagbaw, Vivian Welch

**Affiliations:** 1 Temerty Faculty of Medicine, University of Toronto, Toronto, Ontario, Canada; 2 Bruyère Research Institute, University of Ottawa, Ottawa, Ontario, Canada; 3 School of Medicine, University College Cork, Cork, Ireland; 4 Faculty of Medicine, University of British Columbia, Vancouver, British Columbia, Canada; 5 Department of Psychiatry, University of Toronto, Toronto, Ontario, Canada; 6 Women's College Hospital, Toronto, Ontario, Canada; 7 Faculty of Social Sciences, University of Ottawa, Ottawa, Ontario, Canada; 8 Freelance health research librarian, Ottawa, Ontario, Canada; 9 Hassanah Consulting, Seattle, Washington State, USA; 10 Healthcare Research Associates, LLC/S.T.A.R. Initiative, California, USA; 11 London School of Hygiene and Tropical Medicine, London, UK; 12 London International Development Centre, London, UK; 13 Clinical Epidemiology Program, Ottawa Hospital Research Institute, Ottawa, Canada; 14 Office for Patient Engagement in Research Activity (OPERA), Ottawa Hospital Research Institute, Ottawa, Ontario, Canada; 15 School of Epidemiology and Public Health, University of Ottawa, Ottawa, Ontario, Canada; 16 Dalla Lana School of Public Health, University of Toronto, Toronto, Ontario, Canada; 17 Well Living House, Li Ka Shing Knowledge Institute, Unity Health Toronto, Toronto, Ontario, Canada; 18 Royal College of Physicians and Surgeons of Canada, Ottawa, Ontario, Canada; 19 Centre for Evidence Based Health Care, Department of Global Health, Stellenbosch University, Stellenbosch, South Africa; 20 Department of Evidence and Intelligence for Action in Health, Pan American Health Organization (PAHO/WHO), Washington, DC, USA; 21 Doctoral Programme on Methodology of Biomedical Research and Public Health, Universitat Autònoma de Barcelona, Barcelona, Spain; 22 Department of Public Health and Epidemiology, RCSI University of Medicine and Health Sciences, Dublin, Ireland; 23 Judith Lumley Centre, School of Nursing and Midwifery, La Trobe University, Melbourne, Australia; 24 Ngangk Yira Research Centre for Aboriginal Health and Social Equity, Murdoch University, Perth, Australia; 25 Institute of Health and Wellbeing, University of Glasgow, Glasgow, UK; 26 Department of Health Research Methods, Evidence, and Impact, McMaster University, Hamilton, Ontario, Canada; 27 School of Psychology, University of Ottawa, Ottawa, Ontario, Canada; 28 Cochrane South Africa, South African Medical Research Council, Cape Town, South Africa; 29 Centre for Evidence Based Health Care, Department of Global Health, Stellenbosch University, Stellenbosch, South Africa; 30 School of Health and Human Performance, Georgia College, Milledgeville, Georgia, USA; 31 Centre for Global Child Health, Hospital for Sick Children, Toronto, Canada; 32 Institute for Global Health and Development, Aga Khan University, Karachi, Pakistan; 33 Biomedical Research Institute Sant Pau, Hospital de la Santa Creu i Sant Pau, Barcelona, Spain; 34 Department of Public Health and Sports Science, University of Exeter College of Medicine and Health, Exeter, UK; 35 Family Medicine Department, School of Medicine, Pontificia Universidad Catolica de Chile, Santiago, Chile; 36 Michael G. DeGroote Institute for Pain Research and Care, McMaster University, Hamilton, Canada; 37 Faculty of Health Sciences, School of Rehabilitation Therapy, Queen’s University, Kingston, Canada; 38 World Health Organization Collaborating Centre for Knowledge Translation and Health Technology Assessment in Health Equity, Ottawa, Canada; 39 Department of Family Medicine, Faculty of Health Sciences, Queen’s University, Kingston, Ontario; 40 Campbell Collaboration, Oslo, Norway; 41 Department of Epidemiology and Biostatistics, Kilimanjaro Christian Medical University College, Tanzania.; 42 International Centre for Eye Health, London School of Hygiene and Tropical Medicine, London, UK; 43 University of Ottawa Heart Institute, Ottawa, Canada; 44 C.T. Lamont Primary Care Research Centre, Bruyère Research Institute, Ottawa, Canada; 45 Department of Family Medicine, Schulich School of Medicine and Dentistry, Western University, London, Canada; 46 Department of Public Health, The University of Bamenda, Bamenda, Cameroon; 47 Nutrition and Health Research Group (NHRG), Bamenda, Cameroon; 48 Faculty of Medicine, University of Ottawa, Ottawa, Canada

## Abstract

**Background:**

Observational studies can inform how we understand and address persisting health inequities through the collection, reporting and analysis of health equity factors. However, the extent to which the analysis and reporting of equity-relevant aspects in observational research are generally unknown. Thus, we aimed to systematically evaluate how equity-relevant observational studies reported equity considerations in the study design and analyses.

**Methods:**

We searched MEDLINE for health equity-relevant observational studies from January 2020 to March 2022, resulting in 16 828 articles. We randomly selected 320 studies, ensuring a balance in focus on populations experiencing inequities, country income settings, and coronavirus disease 2019 (COVID-19) topic. We extracted information on study design and analysis methods.

**Results:**

The bulk of the studies were conducted in North America (n = 95, 30%), followed by Europe and Central Asia (n = 55, 17%). Half of the studies (n = 171, 53%) addressed general health and well-being, while 49 (15%) focused on mental health conditions. Two-thirds of the studies (n = 220, 69%) were cross-sectional. Eight (3%) engaged with populations experiencing inequities, while 22 (29%) adapted recruitment methods to reach these populations. Further, 67 studies (21%) examined interaction effects primarily related to race or ethnicity (48%). Two-thirds of the studies (72%) adjusted for characteristics associated with inequities, and 18 studies (6%) used flow diagrams to depict how populations experiencing inequities progressed throughout the studies.

**Conclusions:**

Despite over 80% of the equity-focused observational studies providing a rationale for a focus on health equity, reporting of study design features relevant to health equity ranged from 0–95%, with over half of the items reported by less than one-quarter of studies. This methodological study is a baseline assessment to inform the development of an equity-focussed reporting guideline for observational studies as an extension of the well-known Strengthening Reporting of Observational Studies in Epidemiology (STROBE) guideline.

Wilson and colleagues emphasise that ‘We have to acknowledge that as researchers we have power. We have to use our power and knowledge responsibly. We have to act. That might be acting to resolve difference or acting to ensure accuracy or acting by refusing to follow the status quo. It requires us to use our power as researchers to change ourselves as individuals, but also all of humankind.’ [1].

Health inequities are unfair and unjust inequalities in health that stem from various social determinants of health (e.g. gender, socioeconomic status, and ethnicity, primarily due to structural racism and systems of oppression [2,3]. Addressing these inequities can promote well-being between and within populations [4]. These social determinants of health have been summarised by several frameworks [5–7], including the PROGRESS-Plus framework that outlines factors stratifying opportunities for health. The mnemonic stands for Place of residence, Race/ethnicity/culture/language, Occupation/out of work, Gender/sex, Religion, Education, Socioeconomic status, and Social capital [8]. Additional factors are recognised in the ‘Plus’ component such as individual characteristics (age, disability), features of relationships (e.g. smoking parents), and time-dependent transitions (temporary health disadvantages) [8].

Despite global commitments to address inequities, there remains a need for more empirical research to identify and understand the complex underlying structures of inequities [9]. The lack of rigour in the collection of health equity data hampers the development of health-equitable programs and policies for better overall health. Many authors have urged researchers to prioritise health equity research in policy, health systems, and health services (including organisation, delivery, prioritisation, and implementation), integrating it into primary and secondary research [10–13].

The experiences of health policies, systems, and services by populations experiencing inequities can be captured well in descriptive or analytical research that evaluates a question without intervention [14,15]. This type of research is conducted by means of observational studies, which tend to predominate in health-related research [16]; they can be used to investigate causal relationships in the presence of a control group [17] and provide valuable knowledge to inform health guidelines and policy decisions. For instance, observational research conducted during the coronavirus disease 2019 (COVID-19) pandemic highlighted the vast inequities in society and informed public health responses to the severe acute respiratory syndrome coronavirus 2 (SARS-CoV-2) [18–20].

To improve evidence to inform equity decisions, comprehensive reporting (credible and transparent) of equity-related aspects in research is essential in assessing the reliability, reproducibility, and methodological rigour of studies and enhancing the social value and accountability of the research [21––23]. Reporting guidelines can contribute to meeting these goals by increasing the completeness and transparency of research papers [21–24]. The Strengthening of the Reporting of Observational Studies in Epidemiology (STROBE) reporting guideline is a well-known tool for enhancing the reporting of observational research. While surveys have found high levels of authors who self-reported the use of the STROBE guideline (over 60% of observational study authors) [25], and the guidelines are mandated or endorsed by many journals, the current guidelines do not include guidance for consideration of equity. Indeed, analyses of observational studies have found a persistent lack of integration and reporting of sex and gender in these studies [26–28], potentially explained by a lack of guidance on how to report equity in these studies.

Given that it is not yet standard practice to apply and use an equity lens in reporting observational health research, we intend to extend the STROBE guidelines to assist in reporting equity. As a part of that objective, we evaluated how equity-relevant observational studies describe the characteristics of their samples, design features and analysis, and interpretation of their findings across PROGRESS-Plus factors.

## METHODS

This methodological review is part of a larger project aimed to develop reporting guidelines for equity in observational studies [29]. It follows the guidelines of Preferred Reporting Items for Systematic Reviews and Meta-Analyses (PRISMA-Equity), PRISMA 2020, and Guidance for Reporting Involvement of Patients and the Public [12,30,31] (Tables S1–3 the **Online Supplementary Document**). The protocol for this review has been previously published [32].

### Patient and public engagement

JT and GSR are part of our patient and public advisory board for the development of the STROBE-Equity guidelines. These team members experience inequities and have experience with participating in health equity research. They were recruited by nomination or involvement in previous groups. The patient and public partners were involved in discussions identifying the need for the development of the guidelines. They were involved in refining the STROBE-Equity checklist by identifying areas where they believed equity should be addressed. The patient and public partners were also involved in the design of this methodological assessment and discussions related to highlighting the primary findings. The patient and public partners were regularly updated on this project’s progress through quarterly technical advisory meetings and patient steering committee meetings. The study updates were presented at the meetings and shared through newsletters. Although we shared early iterations of the manuscript with the patient and public partners simultaneously with the researchers via email, we found that this process was not optimal. The methodological comments over-clouded the critical comments and reflections of the patient partners, and this impaired their ability to provide feedback. Therefore, we sent the last iteration to the patient advisors separately, with a separate deadline for turnaround. We found this resulted in better engagement by focusing feedback on which findings were important to populations experiencing inequities and the terminologies used to refer to populations throughout the manuscript.

### Search strategy

An information specialist (TR) developed and peer-reviewed the search strategy using the Peer Review of Electronic Search Strategies guideline [33]. We searched Ovid MEDLINE from January 2020 to March 2022 using a validated search filter for health equity studies [34]. We chose this period because we anticipated a focus on health equity due to the pandemic. We enhanced the filter for observational studies by adding two terms to identify cross-sectional studies [35]. Additional terms such as ‘instrumental variable’, ‘discontinuity design’, ‘interrupted time series’, ‘discontinuity design’, ‘matching’, ‘synthetic control’, and ‘difference-in-difference’ were included to capture observational econometric studies (**Online Supplementary Document**). Review articles and randomised controlled trials were excluded using validated study design filters [36].

### Eligibility criteria

Following our study protocol [32], we purposefully randomly selected 320 health equity-relevant observational studies using Jull and colleagues framework [37] using a three-factor randomised sampling approach to achieve balance across: country income settings; whether or not the study related to COVID-19, and whether the study focusing on population(s) experiencing inequalities.

Detailed explanations of purposive random sampling, sample size determination, and definitions of key terms (context, health equity, health equity-relevant studies and observational studies) can be found in the published protocol [32] and **Online Supplementary Document**.

### Screening and data extraction

We placed the retrieved articles in random order using the DistillerSR, version 2.35 (DistillerSR Inc., Ottawa, Canada) random order generator feature, and we then systematically screened articles in order until we had included the pre-specified number of 320 articles. At the title and abstract stage, studies were screened by one of the project team members (AA, MB, LA, JH, AJ, OD) [38]. Full texts were screened by an investigator within that group (AA, MB, LA, JH, AJ), and researcher OD validated all screening decisions. Conflicts during full-text screening were resolved through discussions among the team members during weekly meetings. We developed and pre-tested a data extraction form using DistillerSR software, which was used to capture study characteristics, including the conditions assessed [39], exposures [40], country, conflict of interest, funding and use of a reporting guideline. A data dictionary was developed and refined through pre-testing to ensure consistent information extraction (**Online Supplementary Document**). We assessed the reporting of health equity considerations across PROGRESS-Plus factors and contextual factors across the whole study using our draft STROBE-Equity checklist [29], including the abstract, background and rationale, population characteristics, results, interpretation of applicability, and discussion).

We analysed the descriptive characteristics of all the included studies using frequencies and percentages. Each equity reporting item was reported as count and percentages with 95% confidence intervals (CIs). We report our findings according to study design elements, analyses, and completeness of reporting. All analyses were conducted using Stata, version 18 (StataCorp LLC, College Station, TX, USA).

## RESULTS

### Search results

**Figure 1:**
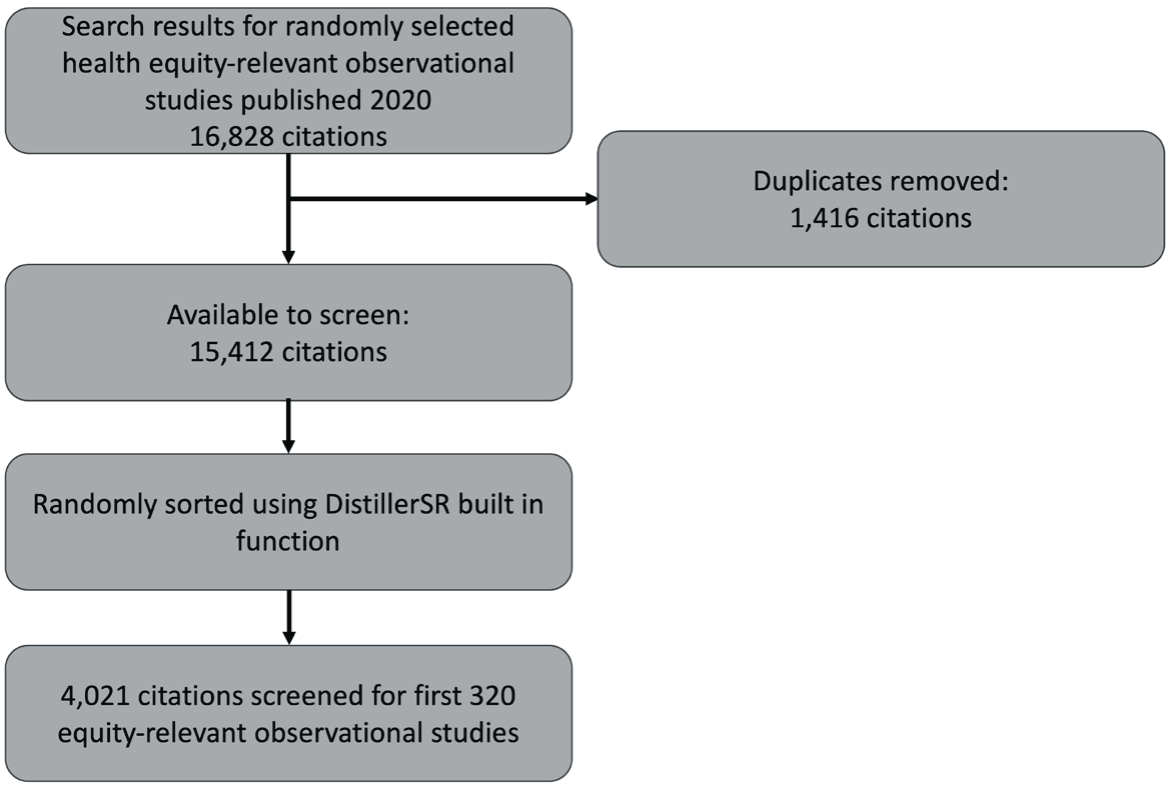
Study flow diagram.

Our search yielded 16 828 articles. After removing duplicates, 15 412 article references remained for the title and abstract screening. A total of 4021 studies were reviewed according to our eligibility criteria to attain our intended sample size of 320 eligible studies (**Figure 1**).

### Characteristics of included studies

Approximately two-thirds of studies (n = 220, 69%) had a cross-sectional design. Half (n = 171, 53%) addressed general health and well-being, while 49 (15%) focused on mental health conditions. In terms of geographical distribution, about one-third (n = 95, 30%) were conducted in North America, 55 (17%) in Europe and Central Asia, 51 (16%) in East Asia and Pacific Asia, and 34 (11%) in South Asia. Further, 128 studies (40%) lacked a specific exposure variable, whereas 121 (38%) assessed health outcomes in relation to specific individual characteristics and behaviours (such as age or ethnicity). Funding for half of the studies (n = 150, 48%) came from governments or not-for-profit organisations. Only 17 studies (5%) reported using the STROBE reporting guidelines for reporting (**Table 1**). The reporting of each STROBE-Equity candidate item as per the drafted STROBE-Equity checklist is presented in Table S7 in the **Online Supplementary Document**.

**Table 1:** Characteristics of included studies (n = 320)

Categories	n (%)
World region	
*East Asia and Pacific*	51 (16)
*South Asia*	34 (11)
*Europe and Central Asia*	55 (17)
*Sub-Saharan Africa*	27 (8)
*Latin America and Caribbean*	29 (9)
*North America*	95 (30)
*Middle East and North Africa*	23 (7)
*Multiple regions*	6 (2)
Study design	
*Cross-sectional*	220 (69)
*Retrospective cohort*	64 (20)
*Prospective cohort*	30 (9)
*Case-control*	6 (2)
Conditions	
*Generic health*	171 (53)
*Mental health*	49 (15)
*Metabolic and endocrine*	16 (5)
*Infection*	13 (4)
*Cardiovascular*	12 (4)
*Cancer and neoplasms*	12 (4)
*Other*	47 (15)
Exposure/intervention	
*Person’s individual characteristics and behaviours*	121 (38)
*Physical environment*	47 (15)
*Social and economic environment*	52 (16)
*No exposure/intervention*	128 (40)
Funding	
*Pharma*	2 (0.6)
*Government/not-for-profit*	150 (47)
*Private*	21 (7)
*No funding*	149 (47)
Conflict of interest	
*Financial*	21 (7)
*Personal*	9 (3)
*Contractual*	7 (2)
*Professional*	3 (1)
Obtained ethics approval	257 (80)
Reported the use of a reporting guideline	17 (5)

### Reporting of health equity considerations in study design aspects

**Figure 2:**
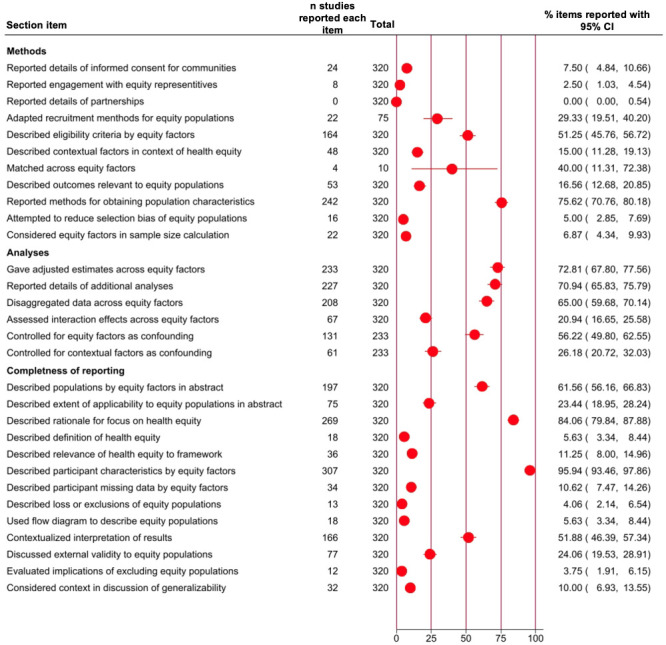
The reporting of equity in equity-relevant observational studies.

Few studies (n = 10, 3%) involved patients, community members or interested groups (also known as stakeholders) in formulating research questions and study design, and none of them provided details on how these partnerships were established or managed during the study (**Figure 2**). A small proportion of the included studies (n = 24, 8%) outlined an informed consent procedure for communities experiencing inequities.

Additionally, about a quarter of the studies (n = 75, 23%) actively recruited participants, with 22 studies (29%) adapting their recruitment methods to reach specific populations defined by PROGRESS-Plus factors. Roughly half of the studies (n = 164, 51%) outlined inclusion or exclusion criteria based on at least one PROGRESS-Plus factor. A minority of the studies (n = 16, 5%) reported efforts to reduce selection bias for populations experiencing inequities, such as using separate inclusion criteria (e.g. cut-offs for clinical indicators) by sex or gender. A subset of the studies (n = 48, 15%) provided contextual information regarding health equity. Further, 10 studies (3%) matched participants across baseline characteristics, four (40%) of which matched participants by at least one PROGRESS-Plus factor. These matching factors were identified by examining the data sets for significant differences across outcomes of interest. A minority of the studies (n = 53, 17%) explained how they determined the relevance of the outcomes to populations experiencing inequities. In 22 studies (7%), authors considered at least one PROGRESS-Plus factor in the sample size calculation.

Three-quarters of the studies (n = 242, 76%) described how the authors of the observational studies obtained information on participant characteristics. The most commonly (n = 85, 35%) collected characteristics using surveys, followed by hospital or tertiary centre databases (n = 80, 33%). Further, 35 studies (14%) relied on self-reporting or self-selection. In 21 studies (9%), participant characteristics were obtained from electronic health records such as Medicare in the USA and the National Health Service in the UK, while another 21 studies (9%) used investigator-observed approaches such as interviews.

The denominator for adapting recruitment methods to recruit populations experiencing inequities is lower than the total number of studies as it represents the number of studies that used active recruitment. The same applies to the denominator for matching across equity factors and adjusting for estimates.

### Reporting health equity considerations in analyses

**Figure 3:**
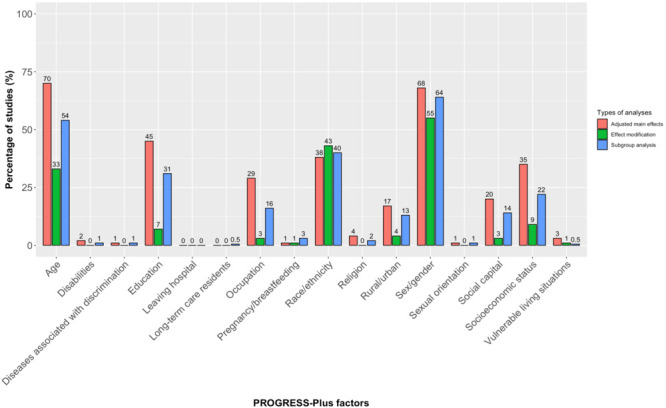
Distribution of analyses in 320 equity relevant-observational studies across PROGRESS-Plus characteristics.

Three-quarters of the studies (n = 233, 73%) examined adjusted associations with health outcomes for at least one PROGRESS-Plus factor. The characteristics analysed varied, with age (n = 164, 70%) and sex (n = 159, 68%) being the most studied factors (**Figure 3**). Conversely, factors like sexual orientation, pregnancy or breastfeeding and diseases linked to discrimination (e.g. HIV) were analysed in only two studies each (1%). Almost all studies (n = 219, 94%) found statistically significant differences in at least one of the analysed PROGRESS-Plus factors.

71% of studies (n = 227) conducted additional analyses by including subgroup analyses or effect modification. Among the included studies, 65% (n = 208) reported subgroup analyses involving at least one PROGRESS-Plus factor. The most used subgroup analysis categories were sex (n = 133, 64%), age (n = 112, 54%), and race or ethnicity (n = 83, 40%). No studies conducted subgroup analyses based on social capital, and analyses involving other additional (Plus) characteristics were reported in less than 5% of the studies.

Moreover, 67 studies (21%) incorporated at least one PROGRESS-Plus factor as an effect modifier. Among these, 63 (20%) conducted effect modification assessments using stratified modelling analysis, which involves stratifying the sample by an independent variable and running a model on each stratum. Stratification was most commonly by sex or gender (n = 28, 42%), while vulnerable living conditions and disabilities were the least analysed (n = 1, 1%). No studies conducted stratified modelling analyses for religion or Plus factors. In 18 studies (6%), effect modification was assessed through interaction terms, with half involving sex or gender. The remaining studies explored the effects of race or ethnicity (n = 6, 33%), age (n = 6, 33%), education level (n = 3, 17%), income level (n = 2, 11%), rurality (n = 1, 6%) and immigration status (n = 1, 6%).

Further, 131 studies (72%) controlled for at least one PROGRESS-Plus factor as a confounding variable in their statistical analyses, and 61 (33%) studies collected and controlled for contextual factors in their research.

### Completeness of equity reporting according to the proposed STROBE-Equity checklist

Over half of the studies (n = 197, 62%) described populations using PROGRESS-Plus factors in abstracts, and 75 (23%) assessed the results’ applicability across PROGRESS-Plus (**Figure 2**). 269 studies (84%) related their rationale to health equity, but only 18 (6%) defined health equity. Further, 36 studies (11%) described the relevance of health equity in exposure theory. The frequency of reporting at least one PROGRESS-Plus factor was 307 (96%) for participant demographics, 36 (11%) for missing data, 13 (4%) for participant loss or exclusion and 18 (6%) for flow diagrams. Overall, half of the studies (n = 166, 52%) contextualised their findings. A quarter (n = 77, 24%) considered applicability across PROGRESS-Plus factors, and 12 studies (4%) reported the implications of excluding people across one or more PROGRESS-Plus factors. 32 studies (10%) discussed contextual factors affecting equity when discussing generalisability (e.g. recruitment of Korean American immigrants from the Korean church as a cultural and social centre may also diminish the influence of socio-economic status and obesity-related health behaviours [41]).

## DISCUSSION

Our study showed that despite many authors recognising equity’s importance, as indicated by over 80% providing a rationale for a focus on health equity, analysis and reporting by PROGRESS-Plus ranged from 0–95%, and almost half of the study design features relevant to equity were reported by less than 25% of articles. This work establishes a baseline assessment for reporting design features relevant to health equity in equity-relevant studies. The observed gaps and shortcomings directly underscore the need for more concerted efforts to address and improve the integration of health equity considerations in research practices. Importantly, these findings directly inform and shape the development of the STROBE-Equity reporting guideline, contributing to the establishment of a robust set of standards for comprehensive reporting of health equity dimensions in future observational studies.

Further, 5% of the studies explicitly mentioned using STROBE guidelines in this sample. A survey of a different sample of observational study authors found that 62% reported using STROBE guidelines [42]. This could suggest that there was over-reporting in that study or that there is less use of STROBE in this equity-focused sample than in that sample. When planning the implementation of STROBE-Equity to improve equity reporting, it will be important to consider how to raise awareness and use of both STROBE and STROBE-Equity amongst relevant audiences of researchers, funders, and journal editors.

This equity reporting assessment might overstate equity considerations in observational research as it focuses on equity-relevant studies which inherently address inequities. A broader analysis of studies could reveal less equity reporting. For instance, in 253 cardiac resynchronisation device studies [26], only 16% considered sex in the study design, and 26% reported sex-related effect sizes. Similarly, in 103 psychiatric studies using routinely collected health data [43], only 14% defined the target population by social determinants and assessed effect modification.

We found relatively few studies conducted in Africa and South America, with most low and middle-income papers from Asia, aligning with findings from a previous assessment of observational studies [44]. This may be a reflection that there are fewer studies published with lead authors from Africa and South America; thus, they had a smaller representation in our sample.

Our study followed a peer-reviewed protocol, ensuring balance across income settings, COVID-19 topics, and equity focus [32]. However, it has limitations. We assessed reported information, not ideal study conduct. Equity relevance judgments relied on abstracts, potentially missing eligible studies with different characteristics. Our search covered 2020–22, so global events like the COVID-19 pandemic and movements like Black Lives Matter may have increased mediated the consideration of equity in studies. Nevertheless, areas for improvement remain.

The relatively high proportion of subgroup analyses across PROGRESS-Plus factors (65%) is explained by the cross-sectional studies included in our sample. Authors frequently disaggregate outcome data by participant characteristics and cross-tabulate them in **Table 1** of cross-sectional study manuscripts (114/220 cross-sectional studies). We found that 21% of studies that assessed PROGRESS-Plus factors as effect modifiers, which is slightly higher than in two previous evaluations of substantive areas (14% in 103 studies of mental health and 13% in 253 studies of heart failure) [26,43] Nonetheless, the reporting of one or more PROGRESS-Plus factor in these studies suggests that there were missed opportunities to explore the role of these factors in effect modification.

Equity considerations in study design and methodology were rarely integrated and reported. Only eight studies reported engaging interest holders experiencing inequities, and just 22 considered equity-related characteristics when determining sample size. Engaging interest holders (alternative term to stakeholders that is more suitable [45]) is a priority supported by leading organisations [46–49] and guidance regarding the matter is improving [50–54]. We recognise the challenges with attaining and sustaining the engagement of members of populations yet advise engaging interest holders, particularly representatives of populations experiencing inequities. It is also a possibility that the engagement of interest holders might have been underreported [55].

The observational studies we included seldom reported efforts to reduce selection bias, like adapting recruitment methods to reach marginalised populations [56,57]. Such adaptations are likely ineffective without engaging these populations in the research. This is especially crucial in studies using routinely collected data. Populations facing social exclusion, such as those experiencing homelessness, substance dependence, sex work, migration, or incarceration, are often excluded or unidentifiable in administrative health data [58,59]. Furthermore, their indicators of social exclusion are not systematically recorded or are inconsistent [59,60]. Failing to consider these factors in observational studies means missing opportunities to generate evidence for these populations.

Mitigating health inequities requires analysing underlying processes like racism and discrimination [61–63]. Observational studies can support this by describing their analytical approach, using logic models, and incorporating health equity in interventions and outcomes. Assessing evidence applicability to populations experiencing inequities is essential for achieving health policy goals, such as the Sustainable Development Goals [14]. Standardising this assessment in all research is crucial, as suggested by our patient advisors.

The findings of this study serve as a baseline assessment for reporting health equity in studies pertaining to equity. The observed gaps and shortcomings directly underscore the need for more concerted efforts to address and improve the integration of health equity considerations in research practices. Importantly, these findings directly inform and shape the development of the STROBE-Equity reporting guideline, contributing to the establishment of a robust set of standards for comprehensive reporting of health equity dimensions in future observational studies. Adherence to these guidelines will enhance the knowledge base regarding the impact of health care practices and policies on health equity and help readers understand what was done and what was found in the research. This will ultimately enhance fairness in the promotion and protection of health.

## CONCLUSIONS

Improving equity data are vital for achieving global goals to ‘leave no-one behind’ [64]. Our study revealed a prevalent recognition of equity’s importance among observational studies published during 2020–23 during which the COVID pandemic was active; however, reporting health equity considerations demonstrates high variability and notable inadequacies. These findings will inform the consensus meeting for our planned STROBE statement equity extension. We are planning an integrated and end-of-grant knowledge translation strategy to disseminate and encourage uptake of STROBE-Equity that is aimed at reaching researchers, funders, research ethics boards and journal editors, all of whom have a role to play in enhancing transparent reporting of health equity in observational studies. Adherence to these guidelines will enhance the knowledge base regarding the impact of health care practices and policies on health equity. This will ultimately enhance fairness in the promotion and protection of health. We invite the scientific community and the public to stay updated on the STROBE-Equity project on our Open Science Framework project page [65].

## Additional material


Online Supplementary Document
