# Supplementary material for: Reporting of equity in observational epidemiology: A methodological review
Source: J Glob Health. 2024 Mar 1;14:04046. doi: 10.7189/jogh.14.04046 (PMC10903926; doi:10.7189/jogh.14.04046)
Supplement: Online Supplementary Document [file jogh-14-04046-s001.pdf]

Supplementary appendix I. PRISMA-Equity reporting checklist

Checklist of Items for Reporting Equity-Focused Systematic Reviews				
**Section**	**Item**	**Standard PRISMA Item**	**Extension for Equity-Focused Reviews**	**Pg #**
**Title**				
**Title**	1	Identify the report as a systematic review, meta-analysis, or both.	Identify equity as a focus of the review, if relevant, using the term equity	1
**Abstract**				
**Structured summary**	2	2. Provide a structured summary including, as applicable: background; objectives; data sources; study eligibility criteria, participants, and interventions; study appraisal and synthesis methods; results; limitations; conclusions and implications of key findings; systematic review registration number.	State research question(s) related to health equity.	4
	2A		Present results of health equity analyses (e.g. subgroup analyses or meta-regression).	4
	2B		Describe extent and limits of applicability to disadvantaged populations of interest.	4
**Introduction**				
**Rationale**	3	Describe the rationale for the review in the context of what is already known.	Describe assumptions about mechanism(s) by which the intervention is assumed to have an impact on health equity.	7
	3A		Provide the logic model/analytical framework, if done, to show the pathways through which the intervention is assumed to affect health equity and how it was developed.	NA
**Objectives**	4	Provide an explicit statement of questions being addressed with reference to participants, interventions, comparisons, outcomes, and study design (PICOS).	Describe how disadvantage was defined if used as criterion in the review (e.g. for selecting studies, conducting analyses or judging applicability).	9, appendix III
	4A		State the research questions being addressed with reference to health equity	9
**Methods**				
**Protocol and registration**	5	Indicate if a review protocol exists, if and where it can be accessed (e.g., Web address), and, if available, provide registration information including registration number.		
**Eligibility criteria**	6	6. Specify study characteristics (e.g., PICOS, length of follow-up) and report characteristics (e.g., years considered, language, publication status) used as criteria for eligibility, giving rationale.	Describe the rationale for including particular study designs related to equity research questions.	11
	6A		Describe the rationale for including the outcomes - e.g. how these are relevant to reducing inequity.	NA

**Information sources**	7	Describe all information sources (e.g., databases with dates of coverage, contact with study authors to identify additional studies) in the search and date last searched.	Describe information sources (e.g. health, non-health, and grey literature sources) that were searched that are of specific relevance to address the equity questions of the review.	10-11
**Search**	8	Present full electronic search strategy for at least one database, including any limits used, such that it could be repeated.	Describe the broad search strategy and terms used to address equity questions of the review.	10-11
**Study selection**	9	State the process for selecting studies (i.e., screening, eligibility, included in systematic review, and, if applicable, included in the meta-analysis).		
**Data collection process**	10	Describe method of data extraction from reports (e.g., piloted forms, independently, in duplicate) and any processes for obtaining and confirming data from investigators.		
**Data items**	11	List and define all variables for which data were sought (e.g., PICOS, funding sources) and any assumptions and simplifications made.	List and define data items related to equity, where such data were sought (e.g. using PROGRESS-Plus or other criteria, context).	11-12
**Risk of bias in individual studies**	12	Describe methods used for assessing risk of bias of individual studies (including specification of whether this was done at the study or outcome level), and how this information is to be used in any data synthesis.		
**Summary measures**	13	State the principal summary measures (e.g., risk ratio, difference in means).		
**Synthesis of results**	14	Describe the methods of handling data and combining results of studies, if done, including measures of consistency (e.g., I^2) for each meta-analysis.	Describe methods of synthesizing findings on health inequities (e.g. presenting both relative and absolute differences between groups).	12
**Risk of bias**	15	15. Specify any assessment of risk of bias that may affect the cumulative evidence		
**across studies**		(e.g., publication bias, selective reporting within studies).		
**Additional analyses**	16	Describe methods of additional analyses (e.g., sensitivity or subgroup analyses, meta-regression), if done, indicating which were pre-specified.	Describe methods of additional synthesis approaches related to equity questions, if done, indicating which were pre-specified	NA
**Results**				
**Study selection**	17	Give numbers of studies screened, assessed for eligibility, and included in the review, with reasons for exclusions at each stage, ideally with a flow diagram.		
**Study characteristics**	18	For each study, present characteristics for which data were extracted (e.g., study size, PICOS, follow-up period) and provide the citations.	Present the population characteristics that relate to the equity questions across the relevant PROGRESS-Plus or other factors of interest.	12
**Risk of bias within studies**	19	Present data on risk of bias of each study and, if available, any outcome level		

		assessment (see item 12).		
**Results of individual studies**	20	For all outcomes considered (benefits or harms), present, for each study: (a) simple summary data for each intervention group (b) effect estimates and confidence intervals, ideally with a forest plot.		
**Synthesis of results**	21	Present results of each meta-analysis done, including confidence intervals and measures of consistency.	Present the results of synthesizing findings on inequities (see 14).	12-16
**Risk of bias across studies**	22	Present results of any assessment of risk of bias across studies (see Item 15).		
**Additional analysis**	23	Give results of additional analyses, if done (e.g., sensitivity or subgroup analyses, meta-regression [see Item 16]).	Give the results of additional synthesis approaches related to equity objectives, if done, (see 16).	NA
**Discussion**				
**Summary of evidence**	24	Summarize the main findings including the strength of evidence for each main outcome; consider their relevance to key groups (e.g., healthcare providers, users, and policy makers).		
**Limitations**	25	Discuss limitations at study and outcome level (e.g., risk of bias), and at review-level (e.g., incomplete retrieval of identified research, reporting bias).		
**Conclusions**	26	Provide a general interpretation of the results in the context of other evidence, and implications for future research.	Present extent and limits of applicability to disadvantaged populations of interest and describe the evidence and logic underlying those judgments.	19
	26A		Provide implications for research, practice or policy related to equity where relevant (e.g. types of research needed to address unanswered questions).	18
**Funding**				
**Funding**	27	Describe sources of funding for the systematic review and other support (e.g., supply of data); role of funders for the systematic review.		

Supplementary appendix II. PRISMA 2020 reporting checklist.

Section andTopic	Item #	Checklist item	Location where item is reported
**TITLE**			
Title	1	Identify the report as a systematic review.	1
**ABSTRACT**			
Abstract	2	See the PRISMA 2020 for Abstracts checklist.	4
**INTRODUCTION**			
Rationale	3	Describe the rationale for the review in the context of existing knowledge.	7
Objectives	4	Provide an explicit statement of the objective(s) or question(s) the review addresses.	7
**METHODS**			
Eligibility criteria	5	Specify the inclusion and exclusion criteria for the review and how studies were grouped for the syntheses.	10
Informationsources	6	Specify all databases, registers, websites, organisations, reference lists and other sources searched or consulted to identify studies. Specify thedate when each source was last searched or consulted.	10-11
Search strategy	7	Present the full search strategies for all databases, registers and websites, including any filters and limits used.	Appendix I
Selection process	8	Specify the methods used to decide whether a study met the inclusion criteria of the review, including how many reviewers screened each recordand each report retrieved, whether they worked independently, and if applicable, details of automation tools used in the process.	11
Data collectionprocess	9	Specify the methods used to collect data from reports, including how many reviewers collected data from each report, whether they worked independently, any processes for obtaining or confirming data from study investigators, and if applicable, details of automation tools used in theprocess.	11
Data items	10a	List and define all outcomes for which data were sought. Specify whether all results that were compatible with each outcome domain in eachstudy were sought (e.g. for all measures, time points, analyses), and if not, the methods used to decide which results to collect.	11-12
	10b	List and define all other variables for which data were sought (e.g. participant and intervention characteristics, funding sources). Describe any assumptions made about any missing or unclear information.	11-12
Study risk of bias assessment	11	Specify the methods used to assess risk of bias in the included studies, including details of the tool(s) used, how many reviewers assessed each study and whether they worked independently, and if applicable, details of automation tools used in the process.	NA
Effect measures	12	Specify for each outcome the effect measure(s) (e.g. risk ratio, mean difference) used in the synthesis or presentation of results.	12

Synthesismethods	13a	Describe the processes used to decide which studies were eligible for each synthesis (e.g. tabulating the study intervention characteristics andcomparing against the planned groups for each synthesis (item #5)).	NA
	13b	Describe any methods required to prepare the data for presentation or synthesis, such as handling of missing summary statistics, or dataconversions.	NA
	13c	Describe any methods used to tabulate or visually display results of individual studies and syntheses.	12
	13d	Describe any methods used to synthesize results and provide a rationale for the choice(s). If meta-analysis was performed, describe themodel(s), method(s) to identify the presence and extent of statistical heterogeneity, and software package(s) used.	NA
	13e	Describe any methods used to explore possible causes of heterogeneity among study results (e.g. subgroup analysis, meta-regression).	NA
	13f	Describe any sensitivity analyses conducted to assess robustness of the synthesized results.	NA
Reporting bias assessment	14	Describe any methods used to assess risk of bias due to missing results in a synthesis (arising from reporting biases).	NA
Certainty assessment	15	Describe any methods used to assess certainty (or confidence) in the body of evidence for an outcome.	NA
**RESULTS**			
Study selection	16a	Describe the results of the search and selection process, from the number of records identified in the search to the number of studies included inthe review, ideally using a flow diagram.	12
	16b	Cite studies that might appear to meet the inclusion criteria, but which were excluded, and explain why they were excluded.	NA
Study characteristics	17	Cite each included study and present its characteristics.	NA
Risk of bias instudies	18	Present assessments of risk of bias for each included study.	NA
Results of individual studies	19	For all outcomes, present, for each study: (a) summary statistics for each group (where appropriate) and (b) an effect estimate and its precision(e.g. confidence/credible interval), ideally using structured tables or plots.	12-16
Results of syntheses	20a	For each synthesis, briefly summarise the characteristics and risk of bias among contributing studies.	NA
	20b	Present results of all statistical syntheses conducted. If meta-analysis was done, present for each the summary estimate and its precision (e.g.confidence/credible interval) and measures of statistical heterogeneity. If comparing groups, describe the direction of the effect.	12-16
	20c	Present results of all investigations of possible causes of heterogeneity among study results.	NA
	20d	Present results of all sensitivity analyses conducted to assess the robustness of the synthesized results.	NA
Reporting biases	21	Present assessments of risk of bias due to missing results (arising from reporting biases) for each synthesis assessed.	NA

Certainty ofevidence	22	Present assessments of certainty (or confidence) in the body of evidence for each outcome assessed.	NA
**DISCUSSION**			
Discussion	23a	Provide a general interpretation of the results in the context of other evidence.	16-18
	23b	Discuss any limitations of the evidence included in the review.	16-18
	23c	Discuss any limitations of the review processes used.	16-18
	23d	Discuss implications of the results for practice, policy, and future research.	16-18
**OTHER INFORMATION**			
Registration andprotocol	24a	Provide registration information for the review, including register name and registration number, or state that the review was not registered.	9
	24b	Indicate where the review protocol can be accessed, or state that a protocol was not prepared.	9
	24c	Describe and explain any amendments to information provided at registration or in the protocol.	9
Support	25	Describe sources of financial or non-financial support for the review, and the role of the funders or sponsors in the review.	19
Competinginterests	26	Declare any competing interests of review authors.	20
Availability of data, code andother materials	27	Report which of the following are publicly available and where they can be found: template data collection forms; data extracted from includedstudies; data used for all analyses; analytic code; any other materials used in the review.	NA

Supplementary appendix III. GRIPP-2 short form checklist

Section and topic	item	Reported on page no
1: Aim	Report the aim of PPI in the study	10
2: Methods	Provide a clear description of the methods used for PPI in the study	10
3: Study results	Outcomes—Report the results of PPI in the study, including both positive and negative outcomes	10
4: Discussion and conclusions	Outcomes—Comment on the extent to which PPI influenced the study overall. Describe positive and negative effects	10
5: Reflections/critical perspective	Comment critically on the study, reflecting on the things that went well and those that did not, so others can learn from this experience	10

Supplementary appendix IV. Electronic search strategies
Database: Ovid MEDLINE(R) ALL < January 1, 2020 to March 14, 2022 >
Search Strategy:
----
1 Residence Characteristics/ (36962)
2 Environment design/ (6967)
3 exp Marital status/ (39193)
4 neighbo?rhood*.mp. (36637)
5 residential environment*.mp. (825)
6 rural*.mp. (187096)
7 inner?city.mp. (56)
8 housing instability.mp. (487)
9 housing insecurity.mp. (250)
10 housing strain.mp. (5)
11 housing security.mp. (58)
12 mortgage problems.mp. (0)
13 foreclosure.mp. (234)
14 eviction*.mp. (879)
15 housing loss.mp. (20)
16 home repossession*.mp. (2)
17 home ownership.mp. (511)
18 (repossess* adj3 hous*).mp. (7)
19 (repossess* adj3 propert*).mp. (1)
20 mortgage delinquency.mp. (8)
21 mortgage arrears.mp. (1)
22 mortgage debt*.mp. (16)
23 overcrowding.mp. (3448)
24 (living adj1 (outside or inside or near* or adjacent)).mp. (4015)
25 (household adj2 size).mp. (1680)
26 (marital status or marriage status).mp. (31349)
27 (widow* or cohabit* or divorce* or single parent* or live* alone).mp. (27787)
28 or/1-27 (330017)
29 Cultural Deprivation/ (1175)
30 Acculturation/ (6795)
31 Culture/ (33971)
32 Cross-Cultural Comparison/ (26976)
33 Cultural Characteristics/ (16839)
34 Cultural Diversity/ (12490)
35 Language/ (46957)
36 "Transients and Migrants"/ (13208)
37 exp "Emigrants and Immigrants"/ (14537)
38 Minority groups/ (16136)
39 Minority health/ (875)
40 Prejudice/ (25326)

41 Racism/ (4861)
42 Xenophobia/ (86)
43 Social Discrimination/ (1512)
44 exp Race Relations/ (7363)
45 exp Ethnic Groups/ (100100)
46 exp Continental Population Groups/ (239045)
47 Refugees/ (11940)
48 minorit*.mp. (92750)
49 migration background.mp. (706)
50 racial.mp. (67973)
51 racism.mp. (9015)
52 ethnology.mp. (173834)
53 race.mp. (127050)
54 ethnic*.mp. (202729)
55 non?English.mp. (24)
56 language other than.mp. (522)
57 latino*.mp. (39069)
58 latina*.mp. (4953)
59 hispanic*.mp. (69106)
60 whites.mp. (87172)
61 caucasian*.mp. (66696)
62 non?white.mp. (3104)
63 Torres Strait Islander.mp. (1924)
64 aboriginal.mp. (9547)
65 native american.mp. (4361)
66 inuit.mp. (1966)
67 eskimo.mp. (725)
68 first nation*.mp. (5593)
69 indigenous.mp. (39952)
70 english as a second language.mp. (477)
71 foreign language.mp. (1343)
72 or/29-71 (836500)
73 Occupations/ (23971)
74 Unemployment/ (7504)
75 occupations.mp. (47174)
76 unemployment.mp. (16338)
77 or/73-76 (63010)
78 exp Gender Identity/ (22007)
79 Women's Health/ (28902)
80 gender differences.mp. (30954)
81 (sex disparit* or sex difference?).mp. (42164)
82 gender identity.mp. (22431)
83 sex role.mp. (1690)
84 wom#n* role?.mp. (661)

85 m#n* role?.mp. (11082)
86 gender* role?.mp. (3671)
87 servicewomen.mp. (126)
88 Sex factors/ (277593)
89 or/78-88 (382983)
90 exp Educational status/ (56742)
91 Education/ (21484)
92 Schooling.mp. (9356)
93 educational status.mp. (59436)
94 (education* adj2 level?).mp. (58306)
95 ((higher or better or worse or less) adj educated).mp. (6295)
96 ((higher or better or worse or less) adj level? of education).mp. (2638)
97 or/90-96 (138106)
98 Religion/ (15348)
99 religi*.mp. (68361)
100 or/98-99 (68361)
101 Social determinants of Health/ (5373)
102 Psychosocial Deprivation/ (2047)
103 Sociological Factors/ (705)
104 Working Poor/ (18)
105 Hierarchy, Social/ (2327)
106 disparit*.mp. (107679)
107 inequalit*.mp. (43059)
108 inequit*.mp. (15173)
109 equity.mp. (23857)
110 deprivation.mp. (94518)
111 gini.mp. (1730)
112 concentration index.mp. (1836)
113 Socioeconomic Factors/ (168362)
114 Social Welfare/ (9582)
115 exp Social Class/ (43998)
116 exp Poverty/ (47750)
117 Income/ (32414)
118 Social class*.mp. (49421)
119 social determinants.mp. (14316)
120 social status.mp. (6634)
121 social position.mp. (1131)
122 social background.mp. (1234)
123 social circumstance*.mp. (1336)
124 socio-economic.mp. (36354)
125 socioeconomic.mp. (242211)
126 sociodemographic.mp. (60210)
127 socio-demographic.mp. (29413)
128 SES.mp. (23827)

129 disadvantaged.mp. (15643)
130 impoverished.mp. (3913)
131 poverty.mp. (66996)
132 economic level.mp. (1219)
133 assets index.mp. (24)
134 income*.mp. (171387)
135 or/101-134 (737831)
136 Social Stigma/ (11121)
137 social capital/ (1383)
138 Social Control, Informal/ (3654)
139 exp Social Support/ (77078)
140 exp Social Environment/ (57919)
141 Trust/ (11546)
142 Social conditions/ (9515)
143 Social isolation/ (15468)
144 Social marginalization/ (570)
145 Anomie/ (468)
146 social participation/ (3104)
147 social exclusion.mp. (2250)
148 (social adj (capital or cohes* or organis* or organiz*)).mp. (8854)
149 (community adj3 (cohes* or participa*)).mp. (38633)
150 ((neighbourhood or neighborhood) adj cohes*).mp. (206)
151 social relationships.mp. (6855)
152 social network*.mp. (24012)
153 collective efficacy.mp. (567)
154 civil society.mp. (2282)
155 informal social control.mp. (122)
156 neighbo*rhood disorder.mp. (274)
157 social disorgani?ation.mp. (240)
158 anomie.mp. (595)
159 social support.mp. (99640)
160 social participation.mp. (5840)
161 trust.mp. (46358)
162 emotional support.mp. (7283)
163 psychosocial support.mp. (4949)
164 community capital.mp. (21)
165 neighbo*rhood cohesion.mp. (201)
166 social influence.mp. (2434)
167 (soci*context* or soci*-context*).mp. (13084)
168 or/136-167 (294833)
169 Health Status Disparities/ (18779)
170 Health Services Accessibility/ (82712)
171 Health Equity/ (2561)
172 health*care disparit*.mp. (21941)

173 health care disparit*.mp. (1774)
174 health status disparit*.mp. (19108)
175 health disparit*.mp. (17245)
176 health inequalit*.mp. (7354)
177 health inequit*.mp. (3424)
178 medically underserved.mp. (8435)
179 or/169-178 (139489)
180 28 or 72 or 77 or 89 or 97 or 100 or 135 or 168 or 179 (2269450)
181 potential determinants.mp. (2046)
182 significant correlates of.mp. (1319)
183 (independent correlates or independent association*).mp. (12191)
184 variables associated with.mp. (13568)
185 determinants of.mp. (177540)
186 factors associated with.mp. (151541)
187 identif* determinants.mp. (2549)
188 (more likely or less likely or just as likely).mp. (327049)
189 risk factors for.mp. (1159255)
190 (significantly related to or significant predictor).mp. (62284)
191 (also adj2 associated with).mp. (72635)
192 (at increased risk or at decreased risk).mp. (41259)
193 association* between.mp. (615980)
194 (positively associated or negatively associated).mp. (78832)
195 differed by.mp. (13934)
196 (were high* amongst or were low* amongst).mp. (1377)
197 (inverse relationship with or inversely associated with or inversely related to).mp. (45308)
198 reverse association.mp. (240)
199 differentially affects.mp. (2482)
200 evidence of a link between.mp. (1824)
201 (significantly adj3 likelihood of).mp. (3024)
202 protective factors for.mp. (17359)
203 (differ* adj2 according to).mp. (32038)
204 (inverse adj2 gradient).mp. (267)
205 (positive adj2 gradient).mp. (453)
206 (negative adj2 gradient).mp. (390)
207 (trends were adj3 across).mp. (1808)
208 (related to adj3 variable*).mp. (25211)
209 (differences were adj3 explained by).mp. (8456)
210 (significant among or no# significant among).mp. (3830)
211 or/181-210 (2351333)
212 180 and 211 (570972)
213 Arctic Regions/ (7137)
214 Ethnopharmacology/ (1731)
215 Health Services, Indigenous/ (3761)

216 Indigenous Peoples/ (799)
217 Indians, North American/ (14876)
218 Inuits/ (4003)
219 exp Medicine, Traditional/ not (Chinese or China).ti,ab,kf. (27288)
220 ((traditional adj1 (medicine* or heal* or food* or health*)) not (Chinese or China)).ti,ab,kf. (18354)
221 Nunavut/ (363)
222 Shamanism/ (235)
223 (Aboriginal? or aborigine? or indian? or indigene* or indigenous* or tribe? or tribal*).ti,ab,kf. (144163)
224 (antiindigenous or colonial or colonialism or marginal or marginalized or marginalization or racial* or racism or stigma* or xenophobia or (historic* adj2 trauma*)).ti,ab. (184236)
225 Eskimo.mp. (725)
226 Half-breed.mp. (31)
227 Maori.mp. or "Native Hawaiian or Other Pacific Islander"/ (13911)
228 Pasifika.mp. (95)
229 Pacific People.mp. (259)
230 Indians, South American/ or Indians, North American/ or Amerindian.mp. (19174)
231 Alaskan Natives/ (620)
232 nomadic.mp. (1289)
233 or/213-232 (383044)
234 212 or 233 (902902)
235 exp cohort studies/ (2310755)
236 cohort$.tw. (735573)
237 controlled clinical trial.pt. (94736)
238 epidemiologic methods/ (31606)
239 limit 238 to yr=1966-1989 (11199)
240 exp case-control studies/ (1294946)
241 (case$ and control$).tw. (557393)
242 Cross-Sectional Studies/ (415072)
243 cross section*.tw. (480792)
244 235 or 236 or 237 or 239 or 240 or 241 or 242 or 243 (3763013)
245 234 and 244 (340947)
246 clinical trial.mp. or clinical trial.pt. or random:.mp. or tu.xs. (6269964)
247 search:.tw. or meta analysis.mp,pt. or review.pt. or di.xs. or associated.tw. (9888400)
248 246 or 247 (13744084)
249 245 not 248 (89007)
250 249 not (exp animals/ not humans.sh.) (88331)
251 limit 250 to yr="2020 - 2022" (15412

Supplementary appendix V. Supplementary methods

Definitions

Health inequity: Health inequity is defined as avoidable and unfair differences in health.

Context: We defined context as a set of external characteristics that surround an implementation effort (not a part of it) and could impact the effects of the intervention. This would include healthcare system characteristics, institutional setting; and the wider sociopolitical, economic, and cultural infrastructure.

Health equity relevant studies: We define health equity relevant as studies that focus on individuals or populations experiencing inequities or studies of mixed populations that analyzed at least one health outcome across one or more PROGRESS factor. We define health outcomes according to the WHO definition, as "changes as in health status that result from the provision of health services". This includes studies focused on populations experiencing inequities such as people who experience homelessness and asylum seekers, as well as studies which present disaggregated results or evaluate outcomes across one or more PROGRESS factors. However, studies which control for PROGRESS in analyses without analyzing differences across the factors are not considered equity relevant. For example, studies of breast cancer in women would not be considered health equity-relevant since women are not disadvantaged in opportunities for health as it relates to breast cancer care. However, if stratified analysis was done to compare mortality of women living in rural vs urban area (or other PROGRESS factors), the study would be considered equity relevant. Similarly, a study of low-income or racialized women accessing breast cancer care would be considered health equity-relevant because they experience different opportunities in obtaining care.

Observational studies: We define observational studies as analytical or descriptive studies evaluating a research question without changing exposure to an intervention. Observational studies are classified in the STROBE reporting guideline into three types: 1) cohort: following an exposed population over time; 2) case-control: comparing exposures between people with a particular disease outcome (cases) and people without that outcome (controls); and 3) cross sectional: assessing all individuals in a sample at the same point in time. Studies conducted using routinely collected data stored in administrative datasets can be classified into these three types of studies.

Sampling strategy

Following consultations with our interdisciplinary team, including researchers, decision-makers, and representatives from the patient and public community, we devised a three-factor randomized sampling approach to ensure balance in our study selection across key phenomena that were agreed upon to influence the reporting of health equity considerations in the studies. This we wanted to ensure their equal representation in our study sample.

We balanced across the following factors:

1. Studies conducted in high-income countries (HIC) and low- and middle-income countries (LMIC) based on the World Bank classification.
2. Studies focused on COVID-19 and those that are not.
3. Studies focused on populations experiencing inequities and those that stratify their analyses.

The decision to balance by country was grounded in the recognition of the pivotal role that context and resources play in LMIC settings, influencing health inequities within LMIC in comparison to HIC. As for choosingCOVID-19 studies as a factor, we effort to balance studies related to COVID-19 or unrelated to it our strategy includes a deliberate given the documented exacerbation of inequities during the COVID-19 pandemic.

Although our sampling strategy is based on three factors, our comprehensive assessment will encompass all aspects of PROGRESS-Plus in the included studies. Consequently, populations experiencing various forms of inequities, such as those with low income, homelessness, migrants, asylum-seekers, and racialized individuals, will be included.

Sample size

To ensure a representative sample for comparable results, we employed a random selection process supported by a sample size calculation for binary outcomes (pertaining to categorical considerations of equity in the studies). We based our calculation on 95% confidence intervals with a margin of ± 6% for observed proportions of 50%, assuming that half of the studies would report at least one PROGRESS characteristic. According to our calculations, a sample of 320 studies would be sufficient for our study and that sample size aligns with similar studies in the literature. We are aware that our study lacks sufficient power for making comparisons across the three prioritized groups; therefore, we analyzed the data combined.

	LMIC		HIC		Total
	**COVID studies**	**non-COVID studies**	**COVID studies**	**non-COVID studies**	
**General population with subgroup analysis for populations experiencing inequities**	40	40	40	40	**160**
**Focused on populations experiencing inequities**	40	40	40	40	**160**
**Total**	**80**	**80**	**80**	**80**	**320**

References:

Whitehead M. The concepts and principles of equity and health. Int J Health Serv. 1992;22(3):429-45.

Vanderkruik, R., & McPherson, M. E. (2017). A Contextual Factors Framework to Inform Implementation and Evaluation of Public Health Initiatives. American Journal of Evaluation, 38(3), 348–359. https://doi.org/10.1177/1098214016670029

Welch VA, Norheim OF, Jull J, Cookson R, Sommerfelt H, Tugwell P, et al. CONSORT-Equity 2017 extension and elaboration for better reporting of health equity in randomised trials. BMJ. 2017;359:j5085.

Jull J, Whitehead M, Petticrew M, Kristjansson E, Gough D, Petkovic J, et al. When is a randomised controlled trial health equity relevant? Development and validation of a conceptual framework. BMJ Open. 2017;7(9):e015815.

Reeves BC, Deeks JJ, Higgins JP, Shea B, Tugwell P, A WG. Chapter 24: Including non-randomized studies on intervention effects. In: Higgins JPT, Thomas J, Chandler J, Cumpston M, Li T, Page MJ, Welch VA (editors). *Cochrane Handbook for Systematic Reviews of Interventions* version 6.2 (updated February 2021). Cochrane, 2021.

Grimes DA, Schulz KF. An overview of clinical research: the lay of the land. Lancet. 2002;359(9300):57-61.

World Health Organization. European Observatory on Health Systems and Policies. Glossary. 2009.

Von Elm E, Altman DG, Egger M, Pocock SJ, Gøtzsche PC, Vandenbroucke JP, et al. Strengthening the Reporting of Observational Studies in Epidemiology (STROBE) statement: guidelines for reporting observational studies. BMJ. 2007;335(7624):806-8.

Benchimol EI, Smeeth L, Guttmann A, Harron K, Moher D, Petersen I, et al. The REporting of studies Conducted using Observational Routinely-collected health Data (RECORD) statement. PLoS Med. 2015;12(10):e1001885.

Supplementary appendix VI. Data extraction dictionary.

Extraction form question	Example
Is the study sample described across one or more PROGRESS-Plus characteristics?	“The sample comprised 418 non-Hispanic white and 154 Hispanic women.”<br><br>**Study Title: Differences in Pelvic Floor Symptoms During Pregnancy Between Hispanic and Non-Hispanic White Women**.
Are the limits or extent of applicability of the findings described across one or more PROGRESS-Plus characteristics?	“Reducing any of the five MetS [Metabolic Syndrome] components, while taking into account the differences found by socio-economic and workplace characteristics, should be one priority for reducing MetS prevalence.”<br><br>**Study Title: Prevalence and determinants of metabolic syndrome in Spanish salaried workers: evidence from 15 614 men and women**
Was health equity considered in framing the reason for the study? (i.e. across at least one PROGRESS-Plus characteristic)?	
Difference in disease burden (includes incidence, prevalence, progression, mortality, etc…)	“However, when trends for White women are compared with trends for women of other races and Hispanic ethnicity, long-standing racial disparities in breast cancer mortality are evident through descriptive analyses of surveillance data with greater mortality declines observed for White women.”<br><br>**Study title: Recent Changes in the Patterns of Breast Cancer as a Proportion of All Deaths According to Race and Ethnicity**
Difference in access (coverage, services, timeliness, and workforce)	“Variations in access to breast cancer care by race and ethnicity have been documented in numerous studies farther along the cancer care continuum including follow-up after an abnormal exam,[9–11] timeliness of initiation of treatment,[12,13] and

	concordance of therapy with recommended guidelines.[14]"<br><br>**Study title: Recent Changes in the Patterns of Breast Cancer as a Proportion of All Deaths According to Race and Ethnicity**
Difference in effects	"Our previous analyses comparing cardiovascular, cerebrovascular, microvascular, and all-cause mortality outcomes by medication adherence status among veterans with diabetes revealed that while many outcomes were improved by higher rates of medication use, adherence did not prevent all targeted outcomes.[12]Moreover, when adjusting for patient characteristics and first-year medication adherence, differences in outcomes were observed in subgroups of this population, including minorities and those residing in different geographical regions."<br><br>**Study Title: Racial and Regional Disparities in Outcomes Among Veterans Initially Adherent to Oral Antidiabetic Therapies: an Observational Cohort Study**
Is equity described in the theory of how the intervention/exposure is expected to work? (e.g. in the clinical pathway or logic model)	"Studies have repeatedly demonstrated that Black patients are diagnosed with higher T and N category disease than non-Black patients, and this likely contributes to inferior outcomes.[4,7,8] Although factors such as genetic predisposition and an increased prevalence of risk factors (tobacco and alcohol) may contribute to racial disparities,[7,9,10] we postulate that differences in access to health care play a major role in racial disparities in cancer outcomes."<br><br>**Study title: Association of Race and Health Care System With Disease Stage and Survival in Veterans With Larynx Cancer**
Was health equity defined in the study?	"Defined as ensuring that "everyone can obtain the health services they need, where and when they need them, without facing heavy financial hardship" (WHO & World Bank, 2015), achieving UHC has been recognized as the cornerstone of the

	health-related United Nations' sustainable development goals (SGDs)."<br><br>**Study title: The Role of a Health Protection Scheme in Health Services Utilization Among Community-Dwelling Older Persons in Ghana**
Were patients/participants/community involved in research questions formulation and study design?	"The Temmy Latner Centre for Palliative Care (TLCPC) is the largest home palliative care program in Ontario, and physicians provide care 24/7 symptom and case management to patients at a variety of stages of advanced illness, within Canada's most diverse urban setting. Physicians work with a multidisciplinary team of care providers in partnership with government-funded allied health-care providers and health service coordination agencies."<br><br>**The Impact of Socioeconomic Status on Place of Death Among Patients Receiving Home Palliative Care in Toronto, Canada: A Retrospective Cohort Study**
When active recruitment methods were used, did the authors use methods to recruit study participants across PROGRESS-Plus?	"Students were invited to participate via personal invitation to their parents and/or guardians."<br><br>**Study title: Gender differences in blood pressure and body composition in schoolchildren ascendants from Amerindian and European**
Did the authors describe inclusion/exclusion criteria across PROGRESS-Plus characteristics?	"We excluded type 2 diabetes related to pregnancy and participants aged < 18 years (n = 16 133) and cases with missing georeferencing information (n = 13776), leaving a total of 137 820 adults in the study."<br><br>**Study title: Regional variation in type 2 diabetes: evidence from 137 820 adults on the role of neighbourhood body mass index**
Did the authors describe the context of the study in relation to health equity?	"In addition to the individual-level predictors, four neighborhood-environmental variables are included to capture contextual effects: Neighborhood fear, Social cohesion, Residential stability, and Structural disadvantage. Consistent with the extant literature (Jones et al., 2014;

	WilsonGenderson & Pruchno, 2013), Neighborhood fear is based on respondents' answers to statements about the safety of their respective neighborhood community, such that a higher averaged score indicates less safety and greater fear. Social cohesion refers to the degree of relational trust with neighbors and the level of intimacy with them (Ahern & Galea, 2011; Elliott et al., 2014)"<br><br>**Study title: Exploring the contingent associations between functional limitations and depressive symptoms across residential context: a multilevel panel data analysis**
For matched cohort studies, did the authors use any PROGRESS-Plus characteristics for matching? How were they determined and what was the rationale?	Males and females were matched for age, race and key comorbidities in order to account for differences in baseline characteristics between the 2 groups.<br><br>**Study title: Sex Differences in Case Fatality Rate of COVID-19: Insights From a Multinational Registry**
Did the authors report that outcomes were identified as relevant and important to populations experiencing inequities?	"The primary outcome was defined to be the location of death: (1) home, (2) acute care, or (3) PCU or residential hospice."<br><br>"This is especially important given that some studies suggest that quality of death at home is higher than that in institutional settings.[10]"<br><br>**Study title: The Impact of Socioeconomic Status on Place of Death Among Patients Receiving Home Palliative Care in Toronto, Canada: A Retrospective Cohort Study**
Did the authors report how population characteristics were obtained? (e.g. age)	"Participants self-reported their gender (woman or man) and all analyses were carried out following the international recommendations on gender in public-health research [27–30]."<br><br>**Study title: Gender Differences in the Association between Physical Inactivity and Mental-Health Conditions in People with Vision or Hearing Impairment**

Did the authors report efforts to reduce selection bias across PROGRESS-Plus?	"Obesity was defined as BMI ≥28 kg/m2 . Central obesity was defined as a waist circumference ≥90 cm in males and ≥80 cm in females, respectively, according to WHO recommendations for Asian adults."<br><br>**Study title: Association of socioeconomic and lifestyle factors with chronic non-communicable diseases and multimorbidity among the elderly in rural southwest China.**
Did the authors report the use of any PROGRESS-plus characteristics in the determination of the study sample size?	"African American and Hispanic households were oversampled at about twice the rate of Whites to account for unequal probability sampling during HRS study recruitment (Fisher & Ryan, 2018; Ofstedal & Weir, 2011)"<br><br>**Study title: Association of Parenthood With Incident Heart Disease in United States' Older Men and Women: A Longitudinal Analysis of Health and Retirement Study Data**
Did the authors plan any analyses to explore differences or similarities in effects across PROGRESS-Plus characteristics?	"The prevalence with confidence intervals of CHD and its risk factors is presented by area (urban/rural), gender and survey period."<br><br>**Study title: Change in prevalence of Coronary Heart Disease and its risk between 1991-94 to 2010-12 among rural and urban population of National Capital Region, Delhi**
Did the authors describe the use of context for analysis?	"We conducted 2 sets of multivariable models, the first minimally adjusted for age, parity, **neighborhood income quintile, and rurality,…"**<br><br>**Study title: Association of Preexisting Disability With Severe Maternal Morbidity or Mortality in Ontario, Canada**
In statistical models, did the authors use any PROGRESS-Plus characteristics for covariate adjustment?	"Candidate sociodemographic and pregnancy-related predictor variables were selected a priori based on our conceptual model of predictors and consequences of preterm birth and SMM (Figure A1) and administratively available variables. Maternal sociodemographic factors from birth certificates included: age at delivery,

	race/ethnicity, educational attainment, delivery payer, and urban or rural residence based on Federal Information Processing Standard (FIPS) county codes."<br><br>**Study title: A population-based study to identify the prevalence and correlates of the dual burden of severe maternal morbidity and preterm birth in California**
Did the authors report missing data related to individuals or contextual factors associated with health inequities?	"The amount of missing data for our measures is small. Age and sex have no missing values. Education is missing for 322 observations (0.2%). The 225 cases missing race information are coded as "other" and 57 missing wealth observations are coded as the mean for the respective wave."<br><br>**Study title: Persistent, Consistent, and Extensive: The Trend of Increasing Pain Prevalence in Older Americans**
Did the authors describe losses or exclusions of participants across PROGRESS-Plus characteristics?	"Attrition analyses reveal that age and baseline anxiety increase the likelihood of nonresponse in subsequent waves, whereas home ownership decreases it."<br>**Study title: Mental health of older widows and widowers: Which coping strategies are most protective?**<br><br>"We excluded…those who identified as Alaskan Native or American Indian or 'Other' Race (n=6,673) due to small numbers"<br><br>**Study title: Racial and Ethnic Disparities in Health of Adults in the United States: A 20-Year National Health Interview Survey Analysis, 1999-2018**
Did the authors present the flow of participants across PROGRESS-Plus characteristics?	"A total 604 students within the age range were included in the enrollment stage. Sixty-four students were excluded according to the exclusion criteria, N = 33 Mapuches and N = 31 from the European group. The remaining students were separated by gender as follow; Mapuches (N = 119, boys, N = 55; girls, N = 64), and European group (N = 421, boys, N = 199; girls, N = 222)."

	**Study Title: Gender differences in blood pressure and body composition in schoolchildren ascendants from Amerindian and European**
Did the authors describe participant characteristics across PROGRESS-Plus reported?	"The 8.5% of BAME is the sum of approximately 2% Bangladeshi, Indian and Pakistani (BIP), and 6.5% of other minority ethnic groups (non-BIP: White Other, Mixed, Black, Asian, and Arab). 55.5% of our sample participants are women."<br><br>**Study title: COVID-19 and mental health deterioration by ethnicity and gender in the UK**
Did the authors present disaggregated data (conducted subgroup analysis) across one or more PROGRESS characteristics?	"Stratified to ethnicity, IHD was the cause for HF among 52.2% of Asian descent patients and among 11.7% of African descent patients, whereas HHD was the cause of HF among 39.3% of African descent patients compared to 12.7% of Asian descent."<br><br>**Study title: Heart Failure Hospitalizations and Risk Factors among the Multi-Ethnic Population from a Middle-Income Country: The Suriname Heart Failure Studies**
Did the study authors present an association of one or more PROGRESS characteristics with the outcome(s) of interest?	"Controlling for other covariates, the odds of reporting loneliness in patients who identified as black or African American was 2.20 [95% confidence interval (CI) = 1.23, 3.94] times the odds of reporting loneliness in patients who identified as white."<br><br>**Study title: Patient loneliness in an urban, underserved family medicine residency clinic: prevalence and relationship to health care utilization**
Did the authors report analysis across levels within one of more PROGRESS factors (i.e. presents findings for men vs women)?	"Among non-Hispanic Blacks (POR: 1.62; 95% CI = [1.30, 2.03]) and Other (POR: 1.40; 95% CI = [1.03, 1.88]) the odds of having IADLs limitations followed a similar pattern when compared with non-Hispanic Whites"<br><br>**Study title: The Role of Ethnic and Racial Disparities in Mobility and Physical Function in Older Adults**

Did the authors consider the context in interpreting findings?	“Without the generous welfare programs that Sweden provides for the old, the observed growth between ages 60 and 83 in the income–mortality association could have been even larger.”<br><br>**Study title: What Levels the Association Between Income and Mortality in Later Life: Age or Health Decline?**
Did the authors describe external validity/generalizability to populations across PROGRESS-Plus characteristics?	“Our findings are based on population-level data from a single large state in the USA and may not generalize to other geographical areas with differing demographics, risk profiles, and health care systems. Nonetheless, California has a diverse population, robust data for childbirth cohorts, and accounts for approximately one in eight of all US births [4].”<br><br>**Study title: A population-based study to identify the prevalence and correlates of the dual burden of severe maternal morbidity and preterm birth in California.**
Did the authors describe the implications of exclusion of people across PROGRESS-Plus as well as differential participation and/or loss to follow-up?	“This dropout has limited the power of the longitudinal analyses. Moreover, nonrespondent analyses revealed that the volunteering respondents at Time 2 were more likely to be male, younger, healthier, and physically active nonrespondents, which might limit the generalizability of the findings.”<br><br>**Study title: Does Social Capital Benefit Older Adults' Health and Well-Being? The Mediating Role of Physical Activity**
Did the authors describe the impact of context in the discussion of generalizability?	“Another unique aspect of Korean American immigrants is that the Korean community may play a role in the preservation of their culture, especially the Korean church. Most of participants in this study were recruited from Korean churches. Korean American women may have an opportunity to

	maintain their traditional Korean lifestyle by regular connection with a Korean community. The role of the Korean church as a cultural and social center may also diminish the influence of SES on obesity-related health behaviors.”<br><br>**Study title: Relationships of Obesity-Related Behavior Patterns with Socioeconomic Status and Acculturation in Korean American Women.**

Supplementary appendix VII. Reporting of equity considerations in 320 equity-relevant observational studies across the STROBE-Equity extension items (N=320).

STROBE item	Item No	Recommendation	Proposed Equity extension item	N (%)
Title and abstract	1	(*a*) Indicate the study's design with a commonly used term in the title or the abstract		
		(*b*) Provide in the abstract an informative and balanced summary of what was done and what was found	Describe population according to PROGRESS-Plus<br><br>Describe extent/limits of applicability to populations of interest across PROGRESS-Plus characteristics	197 (62)<br><br>75 (23)
Introduction				
Background/rationale	2	Explain the scientific background and rationale for the investigation being reported	When applicable, describe the rationale for focus on health equity across PROGRESS-Plus	269 (84)
Objectives	3	State specific objectives, including any prespecified hypotheses		
Methods				
Study design	4	Present key elements of study design early in the paper	Report who was involved, engaged or consulted in study design (e.g., community, industry, government, etc.)	8 (3)

			When applicable, describe the definition of health equity related terms<br><br>Describe how equity is relevant to the logic model or framework used to describe how the intervention/exposure supposed to work	18 (6)<br><br>36 (11)
Setting	5	Describe the setting, locations, and relevant dates, including periods of recruitment, exposure, follow-up, and data collection	Report whether methods of sampling/recruitment were designed to reach populations across relevant PROGRESS-Plus	22 out of 75 (29)
Participants	6	(*a*) *Cohort study*—Give the eligibility criteria, and the sources and methods of selection of participants. Describe methods of follow-up<br>*Case-control study*—Give the eligibility criteria, and the sources and methods of case ascertainment and control selection. Give the rationale for the choice of cases and controls<br>*Cross-sectional study*—Give the eligibility criteria, and the sources and methods of selection of participants	Give inclusion and exclusion criteria across relevant PROGRESS-Plus<br><br>Report context and relationship to health equity<br><br>Report details of partnerships with populations and communities, where applicable	164 (51)<br><br>48 (15)<br><br>No details provided
		(*b*) *Cohort study*—For matched studies, give matching criteria and number of exposed and unexposed<br>*Case-control study*—For matched studies, give matching criteria and the number of controls per case	When applicable, report whether any PROGRESS-Plus factors used for matching, how categories were determined and why	4 out of 10 (40)
Variables	7	Clearly define all outcomes, exposures, predictors, potential confounders, and	Report whether outcomes were identified as relevant and important to populations across PROGRESS-Plus	53 (17)

		effect modifiers. Give diagnostic criteria, if applicable		
Data sources/measurement	8	*For each variable of interest, give sources of data and details of methods of assessment (measurement). Describe comparability of assessment methods if there is more than one group	Report the method of obtaining population characteristics (e.g., self-reported vs database)	242 (76)
Bias	9	Describe any efforts to address potential sources of bias	Report efforts to reduce selection bias across PROGRESS-Plus	16 (5)
Study size	10	Explain how the study size was arrived at	Report whether PROGRESS-Plus characteristics of interest were considered in determining the study size	22 (7)
Quantitative variables	11	Explain how quantitative variables were handled in the analyses. If applicable, describe which groupings were chosen and why	Report how decisions were made about analyses related to PROGRESS- Plus, including whether any categories were defined, and how they were decided<br><br>Report whether dimensions of context were collected for analysis	No explanations provided<br><br>61 out of 183
Ethical concerns			Report details of informed consent and ethical clearance, particularly for populations vulnerable in the context of research	24 (8)

Statistical methods	12	(*a*) Describe all statistical methods, including those used to control for confounding	If PROGRESS-Plus factors used to control for confounding, describe how they were defined and rationale<br><br>Report whether contextual factors were used in adjustment for confounding	131 out of 183; no rationale provided<br><br>61 out of 183
		(*b*) Describe any methods used to examine subgroups and interactions		
		(*c*) Explain how missing data were addressed	When applicable, explain whether missing data was related to individual or contextual factors associated with health inequities	34 (11)
		(*d*) *Cohort study*—If applicable, explain how loss to follow-up was addressed<br>*Case-control study*—If applicable, explain how matching of cases and controls was addressed<br>*Cross-sectional study*—If applicable, describe analytical methods taking account of sampling strategy		
		(*e*) Describe any sensitivity analyses		
Results				
Participants	13	(a)* Report numbers of individuals at each stage of study—eg numbers potentially eligible, examined for eligibility, confirmed eligible, included in the study, completing follow-up, and analysed		

		(b)* Give reasons for non-participation at each stage	Describe the losses and exclusions of participants across PROGRESS-Plus<br><br>Describe non-response/non- participation across PROGRESS-Plus	13 (4)<br><br>0 (0)
		(c)* Consider use of a flow diagram	When applicable, consider using a flow diagram to describe flow of participants across PROGRESS-Plus	18 (6)
Descriptive data	14	(a)* Give characteristics of study participants (e.g. demographic, clinical, social) and information on exposures and potential confounders	Present characteristics across relevant PROGRESS-Plus characteristics	307 (96)
		(b) * Indicate number of participants with missing data for each variable of interest		
		(c)* *Cohort study*—Summarise follow-up time (eg, average and total amount)		
Outcome data	15	*Cohort study*—Report numbers of outcome events or summary measures over time		
		Case-control study—Report numbers in each exposure category, or summary measures of exposure		
		Cross-sectional study—Report numbers of outcome events or summary measures		
Main results	16	(*a*) Give unadjusted estimates and, if applicable, confounder-adjusted estimates and their precision (eg, 95% confidence interval). Make clear which	if applicable, confounder-adjusted estimates and their precision (eg, 95% confidence interval) across PROGRESS-Plus	233 (73)

		confounders were adjusted for and why they were included		
		(*b*) Report category boundaries when continuous variables were categorized		
		(c) If relevant, consider translating estimates of relative risk into absolute risk for a meaningful time period		
Other analyses	17	Report other analyses done—eg analyses of subgroups and interactions, and sensitivity analyses	Report other analyses done across PROGRESS-Plus (e.g. analyses of subgroups and interactions)	227 (71)
Discussion				
Key results	18	Summarise key results with reference to study objectives		
Limitations	19	Discuss limitations of the study, taking into account sources of potential bias or imprecision. Discuss both direction and magnitude of any potential bias		
Interpretation	20	Give a cautious overall interpretation of results considering objectives, limitations, multiplicity of analyses, results from similar studies, and other relevant evidence	Consider importance of context in interpretation of health equity	166 (52)
Generalisability	21	Discuss the generalisability (external validity) of the study results	Discuss external validity to populations across relevant PROGRESS-Plus characteristics, considering issues of possible self- selection, healthy volunteer bias, losses across PROGRESS-Plus	77 (24)

			Consider implications of exclusion of people across PROGRESS as well as differential participation and/or loss to follow-up<br><br>Consider context in discussion of generalizability	12 (4)<br><br>32 (10)
Other information				
Funding	22	Give the source of funding and the role of the funders for the present study and, if applicable, for the original study on which the present article is based		
